# Mechanistic Insights into the Role of Iron, Copper, and Carbonaceous Component on the Oxidative Potential of Ultrafine Particulate Matter

**DOI:** 10.1021/acs.chemrestox.0c00399

**Published:** 2021-03-02

**Authors:** Ion Tacu, Ida Kokalari, Ornella Abollino, Catrin Albrecht, Mery Malandrino, Anna Maria Ferretti, Roel P. F. Schins, Ivana Fenoglio

**Affiliations:** † Department of Chemistry, University of Torino, Torino 10125, Italy; ‡ IUF-Leibniz Research Institute for Environmental Medicine, Düsseldorf 40225, Germany; § Department of Drug Science and Technology, University of Torino, Torino 10125, Italy; ⊥ Istituto di Scienze e Tecnologie Chimiche “Giulio Natta” SCITEC CNR, Via Fantoli 16/15, Milan 20138, Italy

## Abstract

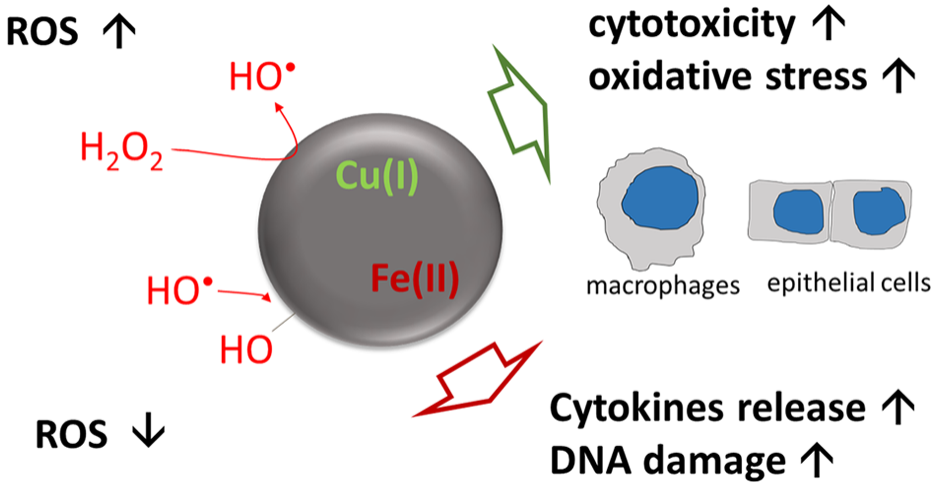

Transition metals play a key role in the pathogenic potential of urban particulate matter (PM). However, air quality regulations include exposure limits only for metals having a known toxic potential like Pb, As, Cd, and Ni, neglecting other transition metals like Fe and Cu. Fe and Cu are mainly found in the water-soluble fraction of PM. However, a fraction of the ions may persist strongly bound to the particles, thus potentially acting as surface reactive sites. The contribution of surface ions to the oxidative potential (OP) of PM is likely different from that of free ions since the redox activity of metals is modulated by their local chemical environment. The aim of this study was to investigate how Fe and Cu bound to carbonaceous particles affect the OP and associated toxicity of PM toward epithelial cells and macrophages. Carbonaceous nanoparticles (CNPs) having well-defined size were loaded with controlled amounts of Cu and Fe. The effect of Cu and Fe on the OP of CNPs was evaluated by electronic paramagnetic resonance (EPR) spectroscopy associated with the spin-trapping technique and correlated with the ability to induce cytotoxicity (LDH, WST-1), oxidative stress (Nrf2 translocation), and DNA damage (comet assay) on lung macrophages (NR8383) and/or epithelial cells (RLE-6TN). The release of pro-inflammatory cytokines (TNF-α, MCP-1, and CXCL2) by macrophages and epithelial cells was also investigated. The results indicate a major contribution of surface Cu to the surface reactivity of CNPs, while Fe has a minor role. At the same time, Cu increases the cytotoxicity of CNPs and their ability to induce oxidative stress and DNA damage. In contrast, surface Fe increases the release of pro-inflammatory cytokines by macrophages. Overall, these results confirm the role of Cu and Fe in PM toxicity and suggest that the total metals content in PM might be a better indicator of pathogenicity than water-soluble metals.

## Introduction

Airborne particulate matter (PM) is composed of particles of both natural and anthropogenic origin. The finest fraction mainly consists of combustion-derived particles formed by organic substances like polycyclic aromatic hydrocarbons (PAHs) and inorganic species (sulfate, nitrate, chloride, ammonium, and metals) adsorbed onto the surface of carbonaceous particles.^1^ The composition of ambient PM is highly variable and complex, depending on many different factors as sources, weather conditions, and topography.^2^ In particular, the kind and the amount of metals are highly variable and depend upon the history of the PM.^3^

The health effects of pollution are today well established.^4^ Exposure to PM strongly correlates with the incidences of severe respiratory disorders (increased hospital admissions for breathing disorders, asthma, emphysema, and chronic bronchitis),^5,6^ with cardiovascular diseases,^5^ and with lung cancer.^7,8^ In addition, air pollution has emerged as a risk factor for neurodevelopmental disorders in children and neurodegenerative disease in adults.^9,10^

Several studies have shown that transition metals are highly associated with PM-induced lung and cardiovascular diseases.^11−17^ However, only metals having known toxic effect are prioritized in air quality regulation. For example, the Ambient Air Quality Directive^18^ that establishes the exposure limit for PM10 requires the Member States to make additional measurements on Pb, As, Cd, and Ni (and benzo(a)pyrene). No exposure limits for other redox-active metals exist.

Iron and copper are ubiquitous in PM.^17,19−22^ These metals can play a major role to the oxidative potential (OP) of PM by direct ROS generation, since they easily undergo redox cycling.^23^

Metals can be typically found in a large amount in the water-soluble fraction of PM,^20,21^ generally referred as the “bioavailable” fraction.^22^ However, part of the metal remains in the insoluble fraction, bound to the particles by coordinative bonds.

Bound metals might have a high pathogenic potential since they are still redox active.^23^ Moreover, their bioactivity may be enhanced since particles can act as carriers of such metals inside cells by a Trojan Horse mechanism. An understanding of the exact role of bound metals using PM samples is not straightforward since cellular uptake, a key process in the toxicity of particulates, is highly dependent upon the size of the particles.^24−26^ Moreover, their OP is poorly predictable based on the reactivity of ions dissolved in water, since it is largely modulated by the coordinative state of the metals. Finally, the carbonaceous fraction of particles can contribute to the overall OP, since it can have both antioxidant^27−29^ and pro-oxidant^30,31^ properties.

In the present study, carbonaceous nanoparticles (CNPs) with a well-defined size (99 nm) have been loaded with controlled amounts of Cu and Fe. The direct OP of pristine CNPs, loaded CNPs, was evaluated by electronic paramagnetic resonance (EPR) spectroscopy associated with the spin-trapping technique and correlated with the ability to induce cytotoxicity (LDH, WST-1), oxidative stress (Nrf2 translocation), and DNA damage (comet assay) on lung macrophages (NR8383) and/or epithelial cells (RLE-6TN). The secretion of pro-inflammatory cytokines (TNF-α, MCP-1, and CXCL2) from the macrophages and epithelial cells was also investigated.

## Experimental Procedures

### Reagents

Sodium polyacrylate, d-(+)-glucose, thionine acetate salt, phosphate-buffered saline powder, and ethylenediaminetetraacetic acid (EDTA) were obtained from Sigma-Aldrich (Germany). 5,5-Dimethyl-1-pyrroline-*N*-oxide (DMPO) was obtained from Cayman Chemicals (USA). Ultrapure water was obtained from a Milli Q Plus system (Millipore, Bedford, MA, USA) and was always used freshly prepared. All other chemicals and solvents used were at least of analytical grade. When not otherwise specified, reagents were purchased from Sigma-Aldrich.

### Synthesis of Glucose-Derived Nanoparticles

Nanoparticles (CNPs) were synthesized from glucose following a protocol previously reported.^29^ A simple and one-step synthesis was followed using the hydrothermal carbonization method. First, glucose (2.0 g) was dissolved in 50 mL of ultrapure water by magnetic stirring, and sodium polyacrylate (15 mg) was then added in order to prevent the typical cross-linking of the nanoparticles during the synthesis. The solution was transferred in a Teflon-lined stainless-steel autoclave (100 mL, Büchi AG), and placed in a preheated oven at 190 °C for 3 h. After the synthesis, the nanoparticles suspension was concentrated and purified with ultrapure water by ultrafiltration using Vivaflow 50R cassettes (Sartorius, 30 kDa cutoff).

### Morphological Characterization

The morphology of the CNPs was evaluated by scanning electron microscopy (SEM, QuantaTM 3D FEG DualBeamTM). The geometric diameter for the CNP samples was measured using the software ImageJ and expressed as the mean of the diameter of up to 600 particles. Electron energy loss spectroscopy (EELS), energy selected image (ESI), and energy-dispersive X-ray analysis (EDX) were performed by a Zeiss LIBRA 200FE-HR TEM operating at 200 kV and equipped with a second-generation in-column Ω filter. The EELS spectra were collected exciting a sample area of about 0.05 μm^2^. Elemental maps of iron were obtained by ESI selecting the electron at the iron L_3_ (708 eV) edge using an energy window as large as 13 eV. The images were processed by means of the iTEM TEM Imaging Platform software (Olympus). The samples were prepared dropping 7 μL of aqueous CNP suspension on a gold grid, covered with a carbon ultrathin film (3–5 nm thick), and let dry overnight.

### Hydrodynamic Diameter and Zeta Potential

The hydrodynamic diameter (dH) distribution and polydispersity index (PdI) of CNPs in Milli-Q water were evaluated using the dynamic light scattering (DLS) technique (Zetasizer Nano Z, Malvern Instruments). A 0.1 mL amount of CNPs suspension was diluted 1:10 in ultrapure water and sonicated by probe sonication (2 min, 30%); the resulting suspension was transferred into a plastic cuvette and analyzed. In order to determine the zeta potential of the NPs, the electrophoretic light scattering (ELS) technique was used (Zetasizer Nano-ZS, Malvern Instruments, Worcestershire, UK). A diluted nanoparticles suspension (0.2 mg/mL) in ultrapure water was sonicated by probe and analyzed. The results are expressed as mean values of three independent experiments.

### Quantification of Surface Acidic Groups Density

A titration assay, previously reported,^29^ was used for quantification of surface acidic groups. Briefly, CNPs (2 mg) were suspended in ultrapure water (1.875 mL) by ultrasonication, and then an aqueous solution of thionine acetate (0.625 mL, 779.2 μM) was added. After 30 min of incubation under magnetic stirring in the dark, the suspension was subjected to centrifugation (11 000 rpm for 30 min). The resulting supernatant was collected and filtered, and its absorbance at 604 nm (Uvikon, Kontron Instruments, Inc., Everett, MA), due to the presence of the nonabsorbed THA, was measured and compared with a calibration curve. The number of surface acidic functionalities was calculated assuming that THA reacts with the acidic groups in a 1:1 stoichiometric ratio. The results are mean values of three experiments.

### Loading of Iron and Copper Ions on NPs Surface

A 4.58 mL amount of Fe(NO_3_)_3_ solution (8.27 mM) or CuSO_4_ solution (31.0 mM) was added to 49 mL of nanoparticle suspension (1.21 mg/mL) and stirred for 15 min. In order to purify the NPs suspension from the metal ions not bonded to the particles, the suspension was centrifuged (11 000 rpm) for 30 min and resuspended in Milli-Q water; the operation was repeated three times.

### Evaluation of the Reduction of Copper and Iron Ions

The reduction of Fe^3+^ ions to Fe^2+^ ions and of Cu^2+^ ions to Cu^+^ by CNPs was evaluated using Ferrozine and bicinchoninic acid (BCA), respectively. In the case of iron, loading was monitored by adding a solution of Ferrozine and ascorbic acid (final concentrations of 1.05 and 0.349 mM, respectively) to the CNP supernatant after incubation of the CNPs with Fe(NO_3_)_3_. The appearance of a purple color indicated the presence of Fe^2+^ ions. To monitor the reduction of copper, 0.61 g of bicinchoninic acid (BCA), 2.7 g of Na_2_CO_3_·10H_2_O, 0.095 g of sodium tartrate dihydrate, and 0.475 g of NaHCO_3_ were dissolved in 50 mL of Milli-Q water. The pH of the solution was adjusted to 11.2 using 1 M NaOH. A 20 μL amount of the solution was mixed to 1 mL of the CNP supernatant after incubation of the CNPs with CuSO_4_ and incubated for 30 min at 37 °C. The appearance of a purple color indicated the presence of Cu^+^ ions.

### Quantification of Loaded Iron and Copper Ions

A 5 mL amount of each NPs suspension (CNP, CNP–Cu, CNP–Fe) was centrifuged (30 min, 11 000 rpm), and the pellet was collected. To each pellet transferred into high-pressure Teflon bombs, 1 mL of H_2_O_2_ (30%) and 4 mL of HNO_3_ ultrapure (>68%) were added. The digestion was carried out using a microwave digestion system (Milestone microwave MLS 1200 Mega). The concentration of the metal ions adsorbed on NPs’ surface was determined by ICP-OES (PerkinElmer Inc., Optima 2000 DV).

### Direct Oxidative Potential

The ability of nanoparticles to generate or scavenge hydroxyl radicals was studied by the EPR/spin-trapping technique (Miniscope MS100, Magnettech, Berlin, Germany).

#### Fenton Reaction

A 0.2 mL amount of nanoparticles suspension (1.11 mg/mL) was transferred into a glass vial with a volume of 5 mL and mixed by stirring. Then a solution (0.25 mL; 0.176 M) of DMPO in water, 80 μL of ultrapure water, and 0.1 mL of phosphate-buffered saline (PBS) (100 mM) was added. In the case of free metal ions experiments, 50 μL of the metal solution (1.00 mM Fe^3+^ and Fe^2+^; 0.73 mM Cu^2+^) was mixed with 0.1 mL of PBS (100 mM), 0.25 mL of DMPO in water (0.176 M), and 0.23 mL of Milli-Q water. The reaction was started by addition of 0.1 mL of a 0.2 M H_2_O_2_ solution. All of the samples were analyzed by EPR after 5, 10, 30, and 60 min of continuous stirring: the synthesis solutions were transferred immediately to a 100 μL glass capillary and analyzed using the EPR spectrometer. The reaction was carried out also in the absence of nanoparticles (negative control). All of the experiments were repeated three times; the obtained results are presented as means ± SDs of AUC calculated after double integration of the spectrum.

#### Hydroxyl Radicals Scavenging

Hydroxyl radicals were generated via Fenton reaction. A 0.2 mL amount of each particle suspension (1.11 mg/mL) was mixed with 0.1 mL of a 100 mM PBS solution and 0.25 mL of DMPO (0.176 M in H_2_O). The mixture was shaken continuously al 37 °C after addition of 80 μL of a FeSO_4_ solution (13 mM) and 0.1 mL of H_2_O_2_ (0.2 M in H_2_O). After 5, 10, 30, and 60 min of incubation, the synthesis solutions were transferred to a 100 μL glass capillary and analyzed using the EPR spectrometer. The reaction was carried out without nanoparticle suspension (only water) to be used as the positive control. The results are presented as means ± SDs of three independent experiments of AUC calculated after double integration of the spectrum.

### Dispersion of Particles in Cell Culture Media

All three stock solutions of the synthesized nanoparticles suspensions were conserved at 4–8 °C. Immediately before cell treatment, particle suspensions were sonicated using an ultrasonic water bath (Bandelin Sonorex, Berlin, Germany) for 10 min, and the CNPs concentration was adjusted to 1.11 mg/mL. For all in vitro experiments the particle suspensions were diluted in Dulbecco’s modified Eagle’s medium (DMEM/F-12 without phenol red, Gibco) supplemented with 1% glutamine and 1% penicillin/streptomycin to obtain the final treatment concentrations. Sterile distilled water was handled in the same way to be used as control. Before addition, the final treatment suspensions were shaken first by a vortex (Bender & Hobein K 550 GE) and then by pipetting up and down to ensure the absence of particle sedimentation.

### Leaching of Metals in the Cell Media

CNP–Cu stock suspensions were diluted in cell culture medium (Nutrient Mixture F-12 Ham containing 1% of glutamine, 1% with penicillin–streptomycin and 5% FBS) in order to obtain a final concentration of 128 μg/mL, chosen among those used for the cellular experiments (see hereafter). Samples were prepared in duplicate and incubated at 37 °C for 24 h. After incubation, the nanoparticles were removed by centrifugation and the amount of Cu in the medium measured by ICP-OES as described above.

### Hydrodynamic Diameter Distribution in the Cell Media

Nanoparticles stock suspensions were diluted in cell culture medium (Nutrient Mixture F-12 Ham containing 1% of glutamine, 1% with penicillin–streptomycin, and 5% FBS) in order to obtain a final concentration of 128 μg/mL, chosen among those used for the cellular experiments. Samples were prepared in duplicate and incubated at 37 °C for 24 h. The dynamic light scattering technique (Zetasizer Nano Z, Malvern Instruments) was used to evaluate the hydrodynamic diameter. Measurements were performed in triplicate for each sample at two time points: 0 and 24 h.

### Direct Oxidative Potential in the Cell Media

Nanoparticles stock suspensions were incubated in cell culture medium (Nutrient Mixture F-12 Ham containing 1% of glutamine, 1% with penicillin–streptomycin, and 5% FBS) at a 1:1 volume ratio for 24 h at 37 °C. Samples were then centrifuged at 11 000 rpm for 30 min. The pelleted nanoparticles were suspended in 100 mM PBS and the ability of the nanoparticles to generate hydroxyl radicals by Fenton reaction evaluated by the EPR/spin-trapping technique as described above. Supernatants were also analyzed in order to investigate the reactivity of the released metal ions. Pristine and loaded CNPs incubated in ultrapure water and a solution of CuSO_4_ (9.26 × 10^–5^ M, corresponding to the amount of Cu released by CNP–Cu in the media after 24h, see hereafter) incubated in cell culture medium were prepared as controls.

### Cells

#### RLE-6TN Epithelial Cells

The rat alveolar type II epithelial cell line RLE-6TN,^32^ hereafter referred to as RLE, was purchased from the American Type Culture Collection (ATCC, USA). These cells were routinely cultured in a 1:1 Nutrient Mixture F-12 Ham mixture purchased from Sigma with 5% fetal calf serum (FCS), 1% of glutamine, and 1% with penicillin–streptomycin (Gibco). They were routinely grown in 75 cm^2^ cell culture flasks at 37 °C and 5% CO_2_.

For the in vitro experiments, cell layers were trypsinized, resuspended in the fresh culture medium, and seeded in sterile 96-multiwell plates or 24-multiwell plates. Since for the RLE cells responsiveness to metals has been reported to decline after high passage numbers,^33^ relatively young cell cultures were used in all of our experiments (<35 passages).

#### NR8383 Macrophages

NR8383 is a cell line (purchased from ATCC, USA) of alveolar macrophages derived from lung lavage of a normal adult male rat. The macrophages were cultured in 75 cm^2^ cell culture flasks with F-12 Nut Mix medium containing 10% FCS, 1% glutamine, and 1% penicillin/streptomycin. Since growing NR8383 cells represent a mixture of suspended and attached cells, both fractions were always collected for routine splitting as well as for seeding prior to experiments. Depending on the experiment performed, the NR8383 were seeded in 96-multiwell plates or 24-multiwell plates fresh culture medium.

### Cell Viability

Cell viability was determined by a colorimetric assay kit based on the water-soluble tetrazolium salt WST-1 (Roche Diagnostics, Germany), which determines the metabolic activity of the cells. Therefore, the RLE cells were seeded with a volume of 100 μL/well in sterile 96-multiwell plates at a density of 10 000 cells per well. The cells were allowed to grow to confluence for 48 h prior to exposure. The NR8383 macrophages were seeded with a cell density of 40 000 cells/well in a 96-well plate, since this cell line grows slower than the RLE-6TN cell line, and grown for a further 48 h until treatment. Samples were run as six replicates. All marginal wells were avoided as it is known that these cells may grow differently. Outer wells of the plate were used as blank controls. For the NR8383 cells, prior to their treatment the macrophages were centrifuged at 12 000 rpm for 10 min, since they adhere loosely to the plate. The growing medium was then gently removed and replaced with experimental exposure medium.

Cells were treated for 24 h with particles concentrations from 0 to 80 μg/cm^2^ (0, 5, 10, 20, 40, and 80 μg/cm^2^ corresponding to 0, 16, 32, 64, 128, and 256 μg/mL) or with FeSO_4_, Fe(NO_3_)_3_, and CuSO_4_ solutions having ions in concentrations comparable with the concentration of metal ions adsorbed on the surface of the nanoparticles used for the treatment. Untreated cells were used as reference values for 100% viability. At the end of the incubation time, two replicates of each test condition were additionally treated with 1% Triton-X for 5 min as positive controls. The reagent WST-1 (10 μL/well) was added, and the plates were incubated for 1 h at 37 °C and 5% CO_2_. The absorbance of the samples was measured at 450 and 630 nm (used as reference wavelength) by a Thermo Multiskan GO Microplate Spectrophotometer. In order to avoid artifacts, the absorbance values were corrected by controls (particles without cells) and Triton-X controls (cells without any metabolic activity). All experiments were repeated at least three times.

### Cell Membrane Integrity

The release of the cytoplasmic enzyme lactate dehydrogenase (LDH) was measured as an indicator of cell membrane integrity. Therefore, 10 000 cells/well (for RLE) and 40 000 cells/well (for NR8383) were uniformly seeded with a volume of 100 μL/well in six 96-well plates. Seeded cells were incubated for 48 h at 37 °C and 5% CO_2_. The resulting plates were treated with a volume of 100 μL/well of all nanoparticle suspensions (concentrations of 0, 5, 10, 20, 40, and 80 μg/cm^2^ corresponding to 0, 16, 32, 64, 128, and 256 μg/mL) with FeSO_4_, Fe(NO_3_)_3_, and CuSO_4_ solutions. Untreated cells were used as reference values for 0% lysed cells. After 24 h of incubation, the NR8383 macrophage plates were centrifuged at 250*g* for 10 min to ensure that the nonadherent macrophages were included in the analysis. For both cell lines, 50 μL/well of supernatant was carefully removed and transferred into new 96-well plates. Then 50 μL of LDH reaction mixture was added to each well, and the plates were then incubated for 20 min at 37 °C and 5% CO_2_. After the incubation time, 25 μL/well of the stop solution (1 N HCl) was added and the absorbance was measured at 490 nm by an ELISA reader (Thermo Multiskan GO Microplate Spectrophotometer). The results were expressed as mean values of three independent experiments.

### Nrf2 Translocation

Expression and translocation into the nucleus of the transcription factor nuclear factor erythroid 2-related factor 2 (Nrf2) was evaluated by immunocytochemistry. Therefore, the RLE cells were seeded into eight 4-chamber culture slides at a density of 62 500 cells/chamber, whereas the NR8383 cells were seeded into 10 wells of two 24-multiwell plates at a density of 250 000 cells/well. Four 4-chamber slides and one 24-well plate were used for Nrf2 identification, and the corresponding 4-chamber slides and 24-well plate were used for Diff-Quick staining. Cells were cultured for 48 h at 37 °C and 5% CO_2_ to allow attaching and confluence reaching. Cells were then treated with CNP, CNP–Fe, and CNP–Cu at concentrations of 5 and 40 μg/cm^2^ and with solutions of Fe(II) and Fe(III) (0.029 mM) and Cu(II) (0.021 mM). After the treatment time, NR8383 cells were scraped from the bottom of the plate, followed by centrifugation (260*g*, 10 min) and a pellet washing step with 1 × PBS (1 mL/well). A 200 μL amount of cell suspension was transferred into a cytospin funnel and spun down at 600 rpm for 5 min using a cytospin centrifuge (Shandon Cytospin 3, Thermo Scientific). For the RLE cells, after separating the chambers from the slides, cells were washed 3 times for 1 min with 1 × PBS. Cells on cytospin slides (NR8383) and chambers (RLE) were fixed in 4% paraformaldehyde/PBS (pH 7.4) for 20 min. Fixed cells were then washed (3 times for 3 min) in PBS, permeabilized with 0.1% Triton in PBS for 5 min, and again washed 3 times for 3 min in PBS. Normal goat serum (dilution 1:65 in PBS) was added (100 μL/chamber or cytospin) for 30 min to prevent nonspecific binding. Then polyclonal Nrf2 antibody (1:50 in PBS) was added, and slides were incubated without cover overnight. All treatment conditions were also stained with a rabbit IgG control (1:50 diluted in PBS) and a PBS control (PBS without primary antibody) to evaluate potential unspecific binding of primary and secondary antibodies. On the second day, unbound antibodies were removed by washing (3 times for 5 min) and an additional staining with the secondary antibody Alexa Fluor 594 Goat-antirabbit IgG (1:200 in PBS) was carried out for 1 h. Finally, after washing three times for 5 min, nuclei were counterstained with 4′,6-diamidino-2-phenylindole (DAPI) (1 mg/mL diluted 1:1000) for 15 min. The last washing step was carried out (3 × 5 min), and 1 drop/chamber of Prolong Diamond Antifading reagent was added. Covered slides were then dried overnight at RT and stored at 4 °C; analyses were carried out using a fluorescence microscope (ZEISS Axio Imager 2, 100×), and pictures were taken. The color of all pictures was equally adjusted using ImageJ software.

### Comet Assay

The alkaline comet assay, also referred to as single-cell gel electrophoresis (SCGE), is a sensitive and relatively simple method for measuring DNA damage in eukaryotic cells and was first described in 1984 by Östling and Johanson.^34^ For the analysis of DNA damage, the RLE cells were seeded with a volume of 1 mL/well in a sterile 24-well plate at a density of 62 000 cells per well and grown for 48 h. Cells were treated for 4 h with a volume of 1 mL/well of CNP, CNP–Fe, and CNP–Cu suspensions (0, 5, and 40 μg/cm^2^) and of iron(II and III) and copper(II) ions solution at two different concentrations (Fe^2+^ and Fe^3+^ 0.029 mM; Cu^2+^ 0.021 mM). The concentrations of the metal ions solutions used correspond to the number of ions adsorbed on the surface of the nanoparticles at a treatment concentration of 40 μg/cm^2^. After treatment, wells were washed 2 times with PBS buffer and trypsinized to allow cell detachment, and cells were resuspended with 0.5 mL/well of medium. Cell suspensions (10 μL/well) were mixed with 120 μL of low melting point (LMP) agarose by slowly pipetting up and down; 120 μL of this mixture was spread over an agarose-coated slide (slide coated with a water solution of 1.5% w/v normal-melting agarose) and covered with a coverslip. All further procedures were performed in a dark room in order to protect DNA from light damage. Once the agar was solidified and coverslips were removed, slides were incubated overnight (refrigerator, 4–8 °C) in the lysis solution (2.5 M NaCl, 100 mM EDTA, 10 mM Tris, 10% DMSO, 1% Triton x-100; pH adjusted to 10 with NaOH) in order to lyse the cell membranes. Slides were then rinsed three times for 5 min each with cold water to remove lysis solution. Then the slides were incubated for 20 min in the electrophoresis tank filled with electrophoresis buffer (0.3 M NaOH, 1 mM EDTA, pH 13) to allow for DNA unwinding. The samples were then submitted to electrophoresis in the same buffer for 10 min at 24–26 V and 280 mA. After electrophoresis, the slides were rinsed three times for 5 min each with neutralization buffer (0.4 M Tris, pH 7.5, adjusted with HCl), followed by 5 min of incubation in 96% ethanol. The slides were then air dried, stained with ethidium bromide (10 μg/mL) diluted 1:5, and visualized in a fluorescence microscope (Olympus BX6A coupled with U-RLF-T UV burner). At least 50 representative images of each slide were acquired at a magnification of 400× and analyzed by Comet Assay IV (Perceptive Instruments, Wiltshire, UK) software.

### TNF-α, MCP-1, and CXCL2 Release

The concentrations of the pro-inflammatory cytokine tumor necrosis factor-α (TNF-α) and the chemokines monocyte chemoattractant protein-1 (MCP-1 and C-X-C Motif Chemokine Ligand 2 (CXCL2) in the cell culture supernatants were quantified by enzyme-linked immunosorbent assay (ELISA) using Quantikine ELISA kits (BioTechne). Therefore, cells were seeded for 48 h into 24-multiwell plates at cell densities of 250 000 cells/well (NR8383) and 62 500 cells/well (RLE). After culture medium replacement, the cells were treated at particle concentrations of 0, 5, and 40 μg/cm^2^ and at metal concentrations of 0.029 mM for Fe^2+^ and Fe^3+^ solutions and 0.021 mM for Cu^2+^ solution. After 24 h of incubation, the supernatants were collected by centrifugation at 250*g* and 4 °C for 5 min. The supernatants were transferred into new sterile Eppendorfs and stored at −80 °C until analysis. ELISAs were performed using the protocol provided by the commercial ELISA kits and analyzed spectrophotometrically (Thermo Multiskan GO Microplate Spectrophotometer).

### Statistical Analysis

Statistical significance of data was calculated by one-way analysis of variance (ANOVA) using Tukey’s pairwise comparison of means. All data expressed as means ± SDs were compared to the control. Significance was ascribed at *p* < 0.05, and all analyses were carried out by SPSS statistics, Version 24 (IBM Corp., USA).

## Results

### Synthesis and Characterization of Nanoparticles

**Figure 1:**
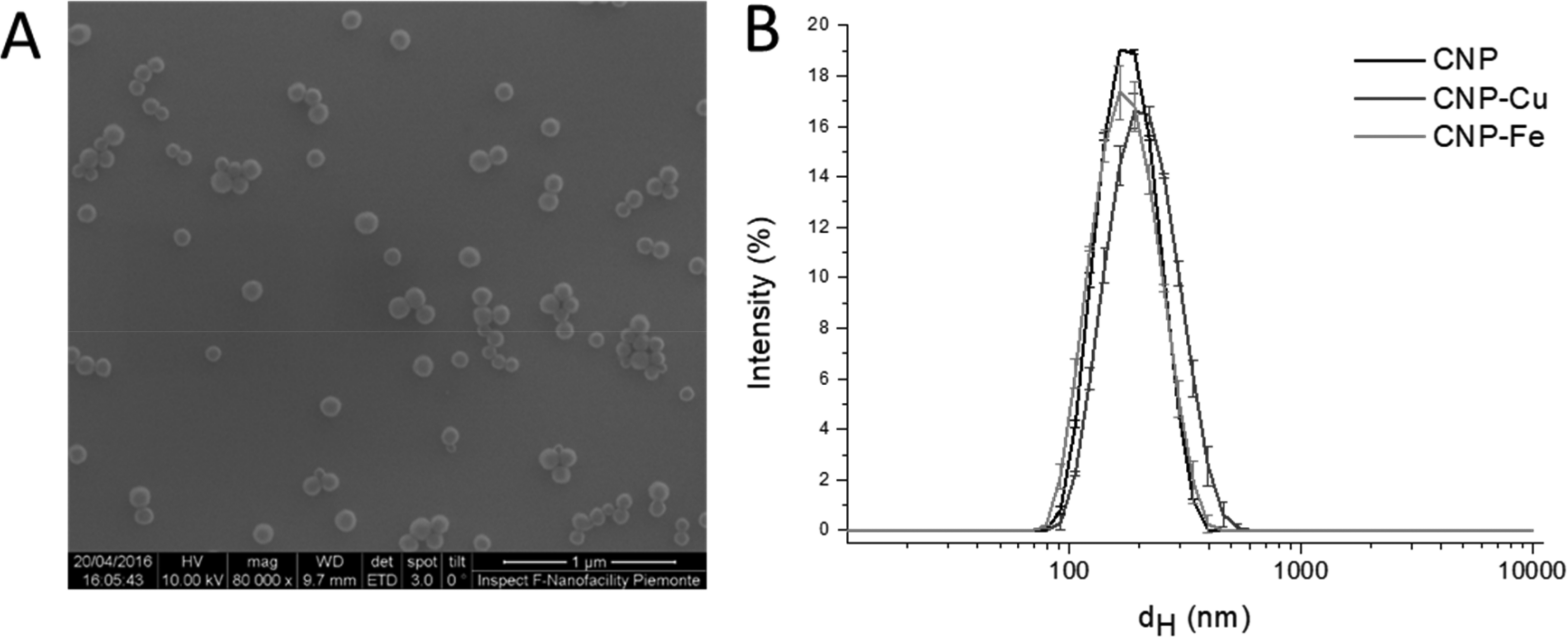
Morphological characterization of CNPs. (A) Representative SEM micrograph of pristine CNPs. (B) Hydrodynamic diameters distribution (% intensity) of pristine and loaded CNPs measured by DLS.

CNPs of defined size and composition were prepared by a hydrothermal synthesis using glucose as precursor, following a protocol previously described by some of us.^29^ This method of synthesis leads to the production of perfectly spherical nanoparticles, as shown in Figure 1A. The particles are composed of amorphous carbon with graphitic patches pervasive in the amorphous framework with a structure close to carbon black^29^ and soot.^27^ The size distribution of the nanoparticles was assessed by SEM and dynamic light scattering (DLS) (Figure 1B) and confirmed by TEM (Figure S1A). The mean geometrical diameter was 99.62 ± 25.21 nm, the mean hydrodynamic size (number) was 166.9 ± 6.3 nm, and the polydispersity index (PdI) was 0.098 ± 0.05 (Table S1), which corresponds to a narrow size distribution range.

Pristine nanoparticles (CNPs) were loaded with Fe(III) (CNP–Fe) or Cu(II) (CNP–Cu) by incubating the particles with soluble salts and removing the ions weakly or not adsorbed at the surface by washing. The amount of metals bound onto the nanoparticles was quantified by inductively coupled plasma-optical emission spectrometry (ICP-OES). In Table 1 the results of the analysis are reported.

**Table 1:** Amount of Metals Loaded onto CNPs Measured by ICP-OES and ζ Potential

sample	mg metal/g NPs	no. of ions/SA (ions/nm^2^)	surface acidic groups/metal ions ratio	ζ pot (mV)
CNP				–53.9 ± 0.651
CNP–Fe	12.60	3.00	0.8	–43.7 ± 0.231
CNP–Cu	10.39	2.18	1.1	–39.5 ± 0.608

The quantity of loaded metals (around 10 mg/g) and the surface density were comparable in both samples. Moreover, it was in the same order of magnitude of the typical content of metals in environmental PM samples.^36^ Considering that a single CNP is decorated by 4 × 10^4^ acidic groups with a density of 3 groups/nm^2^,^29^ the number of acidic groups per loaded metal ion was also calculated (Table 1). A mean of one metal ion/acidic group was present on the surface of the CNPs.

A very weak L_3,2_ EELS signal of iron (Figure S1B) was detected on the CNP–Fe sample. This suggests a uniform distribution of iron on the NP surface, as confirmed by ESI iron maps. The EELS technique did not allow the detection of copper in CNP–Cu. This was expected since the signal of copper is weaker than that of iron, suggesting again a uniform distribution of the ions on the NP surface.

During the synthesis of CNP–Fe, the loading process was monitored on the supernatant using a strong chelating agent of Fe(II) ions (Ferrozine) in the presence of ascorbic acid as reducing agent. As expected, the intense purple color of the Ferrozine–Fe(II) complex was observed. However, the same color was unexpectedly observed also in the absence of ascorbic acid (SI, Figure S2), suggesting that particles are able to reduce the iron ions. The possible reduction of Cu(II) ions to Cu(I) was also evaluated using bicinchoninic acid as probe, which selectively binds Cu(I) (Figure S2). The reduction of the ions was observed also in this case.

Suspensions of pristine and loaded nanoparticles in water were analyzed by dynamic light scattering (DLS) (Figure 1A). The three samples exhibit a similar size distribution, with a mean hydrodynamic dimeter in the range of 140–180 nm (in number) and a polydispersity index (PdI) lower than 0.1 (Table S1), showing that the metals do not induce agglomeration of the particles or modification of the particles morphology, as confirmed by TEM (Figure S1).

As expected, the zeta potential of the different CNPs in water was negative (Table 1),^19^ as a consequence of the negative surface due to the presence of acidic carboxylic groups. Loaded nanoparticles exhibited a slightly less negative zeta potential, confirming the presence of positive ions coordinating a fraction of the surface carboxylic groups.

### Effect of Metals on the Oxidative Potential of Particles

**Figure 2:**
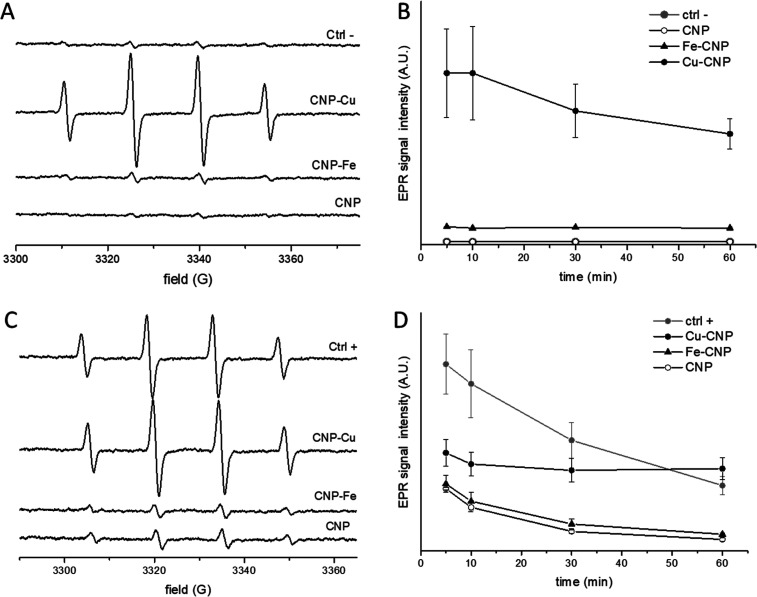
Effect of Cu and Fe on the oxidative potential of CNPs. (Top) Generation of ROS by Fenton reaction. (A) Representative EPR spectra recorded after 60 min following incubation of pristine and metal-loaded CNPs (0.30 mg/mL) in 60.3 mM DMPO, 27 mM H_2_O_2_ in 13.3 mM PBS, pH 7.4. (B) Intensity of the EPR signals vs time. (Bottom) Scavenging activity toward hydroxyl radicals. (C) Representative EPR spectra recorded after 60 min following incubation of pristine and loaded CNP (0.30 mg/mL) with 60.3 mM DMPO, 27 mM H_2_O_2_, and 1.4 mM FeSO_4_ in 13.3 mM PBS, pH 7.4. (D) Intensity of the EPR signals vs time.

The oxidative potential was evaluated by measuring the ability of the particles to generate hydroxyl radicals in the presence of hydrogen peroxide (Fenton-like reaction) and to act as scavengers of hydroxyl radicals. Both properties were studied by EPR spectroscopy coupled with the spin-trapping technique using DMPO as the spin-trapping agent (Figure 2).

Both loaded CNPs incubated with H_2_O_2_ exhibited the typical four peaks in the EPR spectra with a relative intensity of 1:2:2:1, indicating formation of hydroxyl radicals (Figure 2A). However, while CNP–Fe showed a signal only slightly more intense than the control, a much more intense signal was observed in the presence of CNP–Cu, indicating the generation of a large amount of HO^•^ radicals (Figure 2B). However, the intensity of the signal obtained with Cu–CNP was lower than those obtained with free Cu(II) ions at a similar concentration (SI, Figure S3). As expected, the signal obtained with free ions decreased during the time of incubation, with kinetics compatible with the progressive decomposition of the DMPO/HO^•^ adducts into diamagnetic species.^35^ Conversely, the signal intensity obtained with CNP–Cu was nearly constant during this time, suggesting a sustained generation of hydroxyl radicals, with a reaction rate similar to that of the DMPO/HO^•^ adduct decomposition.

The scavenging activity of the particles toward HO^•^ was also evaluated (Figure 2C and 2D) by generating hydroxyl radicals by Fenton reaction in the absence or presence of the NPs. All of the particles modified the concentration of the hydroxyl radicals. However, while for Fe–CNP the signal decreased similarly to what was observed with the pristine ones, suggesting significant scavenging activity, in the presence of CNP–Cu the signal remained high and constant during this time, suggesting the occurrence of the competitive generation of hydroxyl radicals by copper ions.

### Leaching of Metals in the Cell Medium

After loading, the CNPs were washed to remove the Cu and Fe ions weakly adsorbed at the surface. Therefore, the remaining ions are not expected to be released in water. However, in cell media the presence of molecules (e.g., amino acids or carbohydrates) able to coordinate ions might induce the leaching of ions in the solution. In the present case, the amount of iron released in DMEM after 24 h was very low (7.71% of the total amount loaded onto NPs surface), while copper was released to a large extent (73.55%) but with a significant fraction remaining still bound to the particles.

### Oxidative Potential of Particles in the Cell Medium

**Figure 3:**
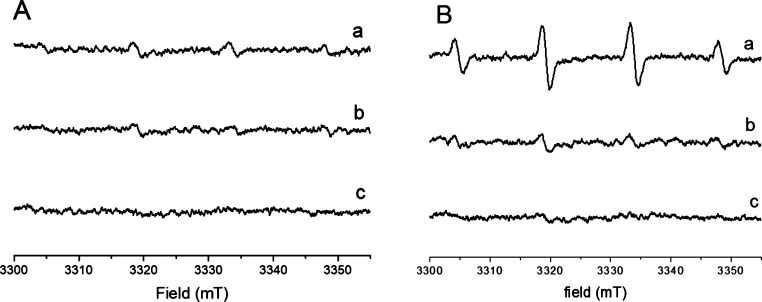
Oxidative potential of loaded CNPs in cell medium. (A) CNP–Fe and (B) CNP–Cu. Representative EPR spectra recorded after 60 min following incubation of (a) nanoparticles incubated in ultrapure water and recovered by centrifugation, (b) nanoparticles incubated in F-12 Ham containing 1% of glutamine, 1% with penicillin–streptomycin, and 5% FBS (0.128 μg/mL) and recovered by centrifugation, and (c) supernatant after removal of nanoparticles: in a solution containing 60.3 mM DMPO, 27 mM H_2_O_2_ in 13.3 mM PBS, pH 7.4.

The oxidative potential was also measured after incubation of the nanoparticles in the cell culture medium for 24 h (Figure 3).

Pristine nanoparticles (data not shown) and CNP–Fe (Figure 3A) exhibit negligible Fenton-like reactivity, similarly to what was observed in water. In the case of CNP–Cu (Figure 3B), significant reactivity was observed, albeit lower than that observed in water. The oxidative potential of the cell medium after incubation with CNP–Cu was also investigated (Figure 3) to monitor the reactivity of leached ions. No reactivity was found. The reactivity of free Cu(II) ions in cell culture medium was investigated for comparison. As shown in Figure S4, in the cell medium the ions lose their ability to generate hydroxyl radicals.

### Size Distribution of Nanoparticles in Cell Culture Medium

The size distribution of the nanoparticles might change in cell media due to the interaction of the nanoparticles with the media components. To monitor such changes, the hydrodynamic diameter was measured for the nanoparticle suspension in the cell medium at a concentration of 128 μg/mL at 0 and 24 h (Figure S5). The analysis evidenced the presence of a single population of nanoparticles for all samples. The suspensions were stable for 24 h. No significant changes in the mean hydrodynamic diameter for pristine and CNP–Fe samples were observed with respect to water, while an increase of the mean hydrodynamic diameter was observed for CNP–Cu. However, the size of the particles remained under 1 μm.

### Effect of Nanoparticles and Metals on the Viability and Membrane Integrity of Lung Macrophages and Epithelial Cells

**Figure 4:**
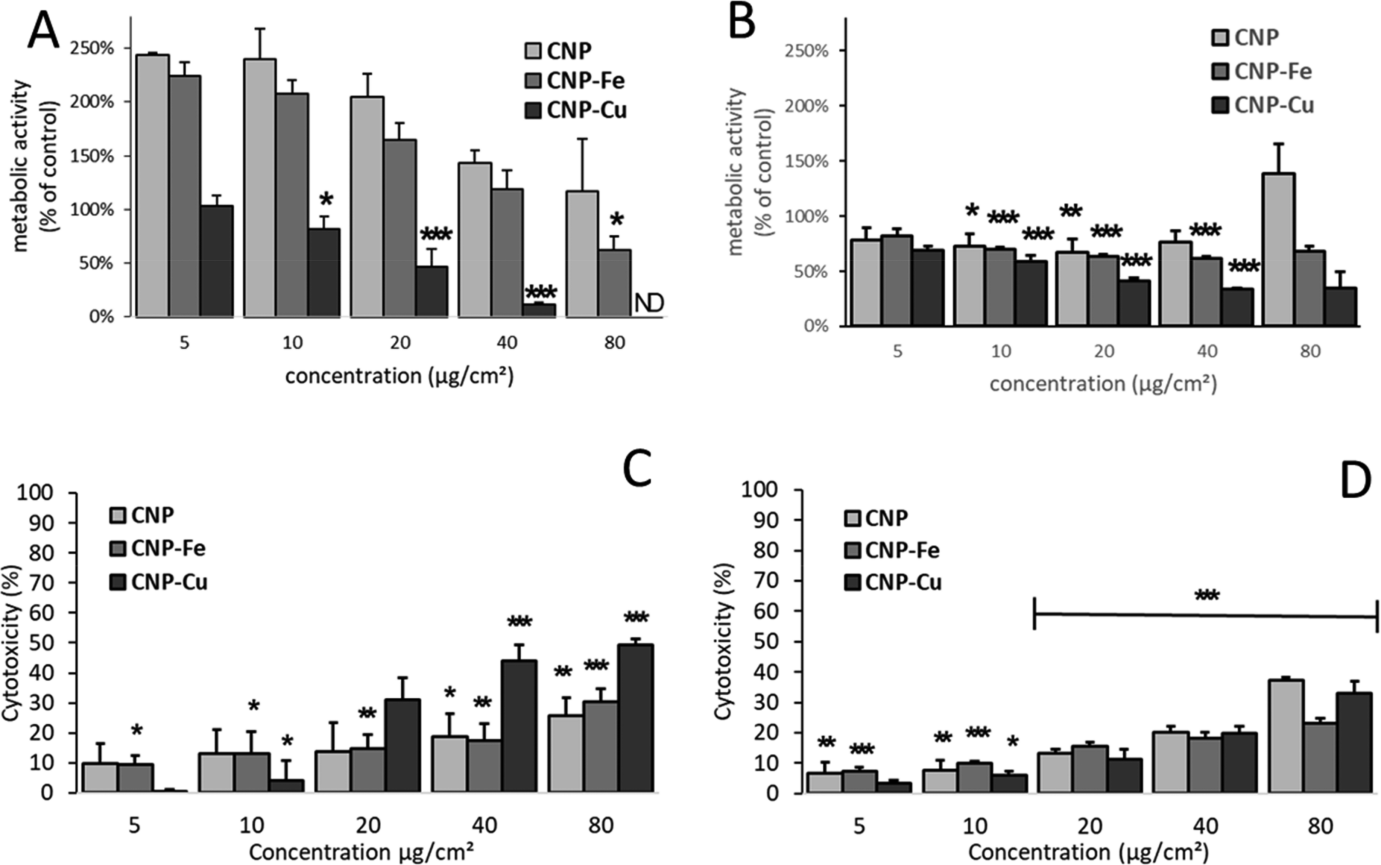
Effect of Cu and Fe on CNPs cytotoxicity toward macrophages and epithelial cells. Effect of pristine and loaded CNP on (A) metabolic activity of NR8383 macrophages (WST-1 assay), (B) metabolic activity in RLE epithelial cells, (C) cell membrane integrity of NR8383 macrophages (LDH assay), and (D) membrane integrity of RLE epithelial cells. * *p* < 0.05, ** *p* < 0.01, *** *p* < 0.001 vs ctrl (0 μg/mL).

NR8383 macrophages and RLE-6TN epithelial cells were treated for 24 h with the pristine and the metal-loaded CNPs in the concentration range of 5–80 μg/cm^2^ , and the viability was subsequently determined by measurement of the mitochondrial activity (WST-1 assay) (Figure 4A and 4B).

Unexpectedly, a significant increase in WST-1 activity with respect to the control was observed when the macrophages were exposed to pristine nanoparticles. A similar effect was observed for CNP–Fe that, compared to the untreated control, was not toxic, except for at the highest concentration (Figure 4A). Conversely, CNP–Cu showed a decrease of viability in a clear concentration-dependent manner. At the same time, CNP–Cu induced a marked dose-dependent damage to the macrophage cell membrane (Figure 4C).

Conversely to what was observed on macrophages, the CNPs slightly reduced the viability of the RLE cells. However, at the highest concentrations (40–80 μg/cm^2^) the CNP induced an increase in WST-1 similarly to what was observed on macrophages (Figure 4B). Both CNP–Fe and CNP–Cu were significantly toxic in a dose-dependent manner with a more evident effect after exposure to CNP–Cu. Both pristine and loaded CNPs induced similarly mild damage to the cell membrane (Figure 4D)

The effect of loaded CNPs on the cell viability was also compared with the effect of soluble salts (Figure S6, SI). Cu–CNP showed a higher toxicity when compared with free ions in both cell lines. In contrast, in the RLE cells, Fe–CNPs were less toxic than free Fe ions in both oxidative states.

### Oxidative Stress Induction in Macrophages and Epithelial Cells

**Figure 5:**
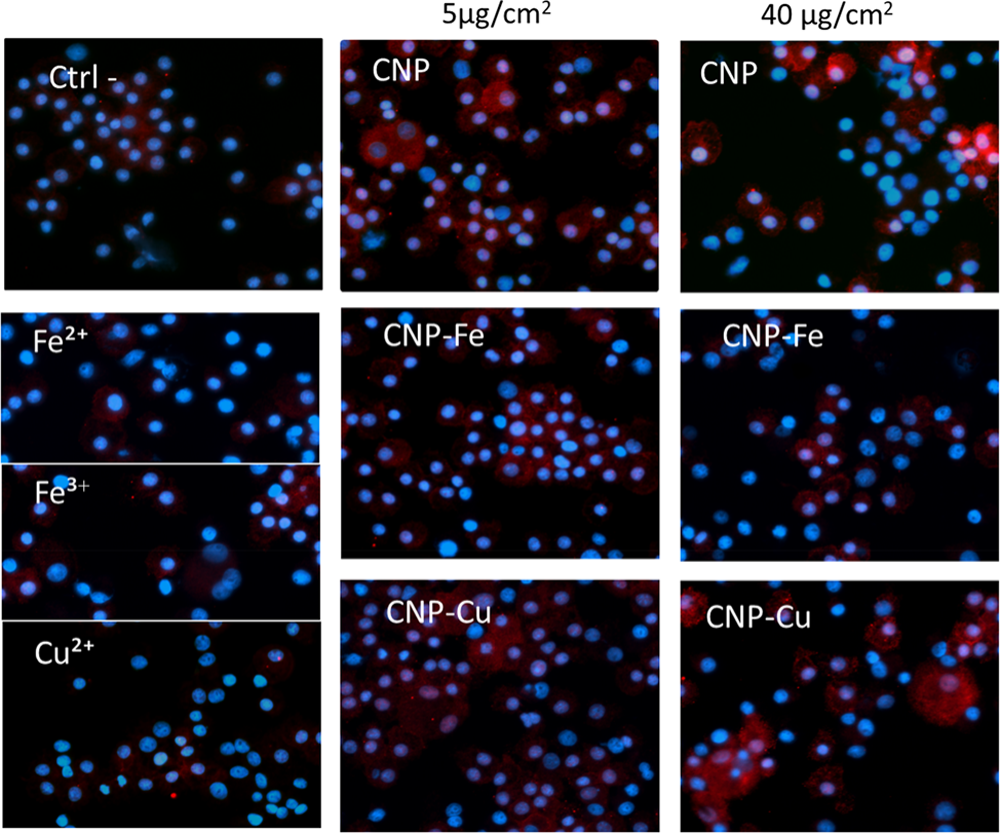
Effect of Cu and Fe on the nuclear translocation of the transcription factor Nrf2 induced by CNPs in macrophages. Representative confocal microscopy images are shown for NR8383 cells treated with 5 or 40 μg/cm^2^ of pristine CNPs, loaded CNPs, or aqueous ions (concentration equivalent to the metal amount loaded onto CNPs at 40 μg/cm^2^ concentration): Nucleus (blue, DAPI); Nrf2 (red).

**Figure 6:**
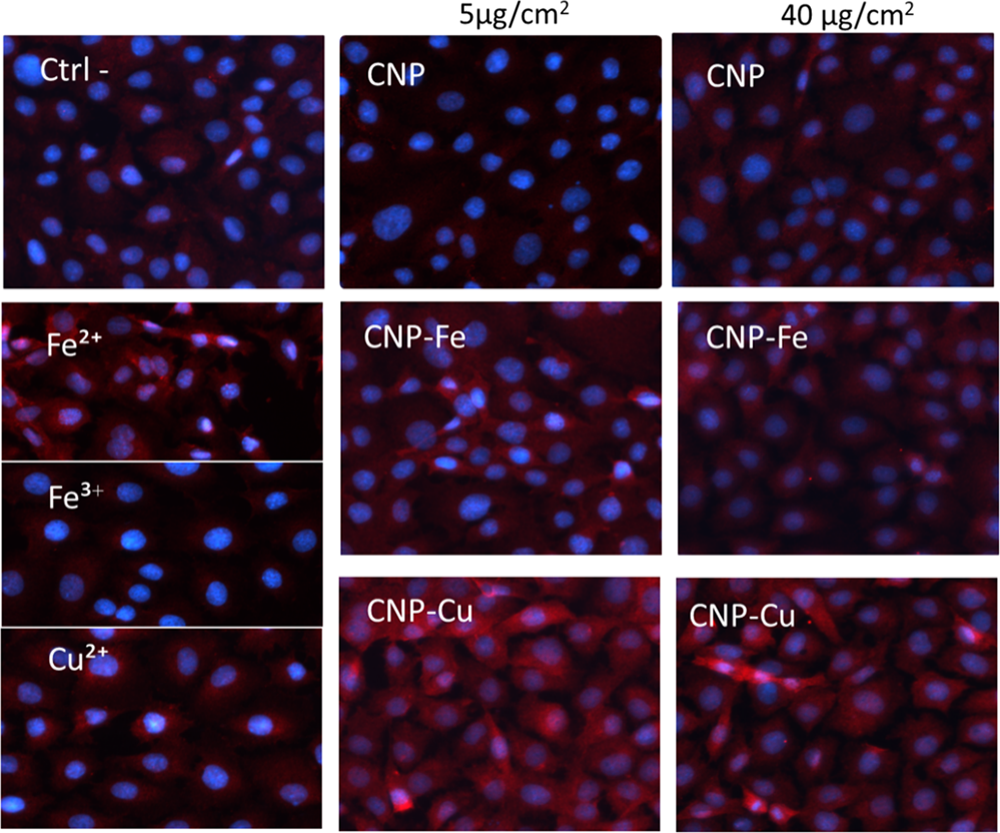
Effect of Cu and Fe on the nuclear translocation of the transcription factor Nrf2 induced by CNPs in epithelial cells. Confocal microscopy images of the RLE cells treated with 5 or 40 μg/cm^2^ of pristine or loaded CNPs and aqueous ions (concentration equivalent to the metal amount loaded onto CNPs at 40 μg/cm^2^ concentration): Nucleus (blue, DAPI); Nrf2 (red).

The ability of the sample to induce oxidative stress in macrophages and epithelial cells was evaluated by measuring the translocation of the transcription factor Nrf2, a marker of activation of antioxidant defenses, into the nucleus. The translocation of Nrf2 into the nucleus may be revealed by confocal microscopy as red fluorescence (Figures 5 and 6).

All of the particles were found to be capable of inducing Nrf2 expression in the cells and in part to induce Nrf2 translocation into the nucleus. However, CNP–Cu-treated cells showed a stronger effect than CNP–Fe and CNP, especially in the RLE cells. No clear effect was observed after treatment of macrophages with soluble metals, while in the RLE cells, translocation was observed for Fe(II) and Cu(II).

### DNA Damage on Epithelial Cells

**Figure 7:**
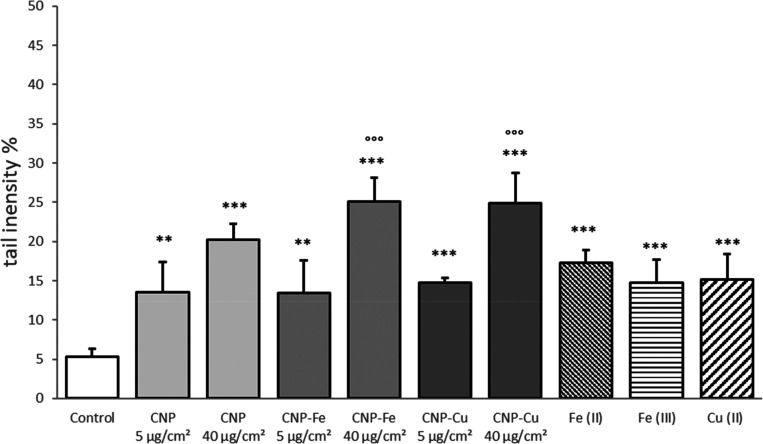
Effect of Cu and Fe on DNA damage induction by CNPs in epithelial cells. DNA damage induced by loaded or pristine CNPs and aqueous ions on RLE epithelial cells following 4 h of treatment. CNP concentration: 5 and 40 μg/cm^2^. Aqueous ion concentration is equivalent to the metal content at 40 μg/cm^2^ concentration. Tail size was measured by the Comet Assay IV (Perceptive Instruments, Wiltshire, UK) software. Data are expressed as the mean of 50 measurements ± SD. ** *p* < 0,05, *** *p* < 0.01 vs ctrl (0 μg/mL); °°° *p* < 0.01 vs aqueous ions.

In order to evaluate the effect of metals on DNA damage, the RLE cells were treated 4 h with 5 or 40 μg/cm^2^ of pristine, loaded particles, or soluble salts, and the DNA damage was assessed by comet assay (Figure 7).

At a concentration of 5 μg/cm^2^, both pristine and loaded nanoparticles showed a similar moderate induction of DNA damage, while at a concentration of 40 μg/cm^2^, the effects of CNP–Fe and CNP–Cu were more pronounced than those of the pristine nanoparticles. Both CNP–Fe and CNP–Cu caused more DNA damage when compared to soluble metals at the same concentration.

### Pro-Inflammatory Chemokines Secretion

**Figure 8:**
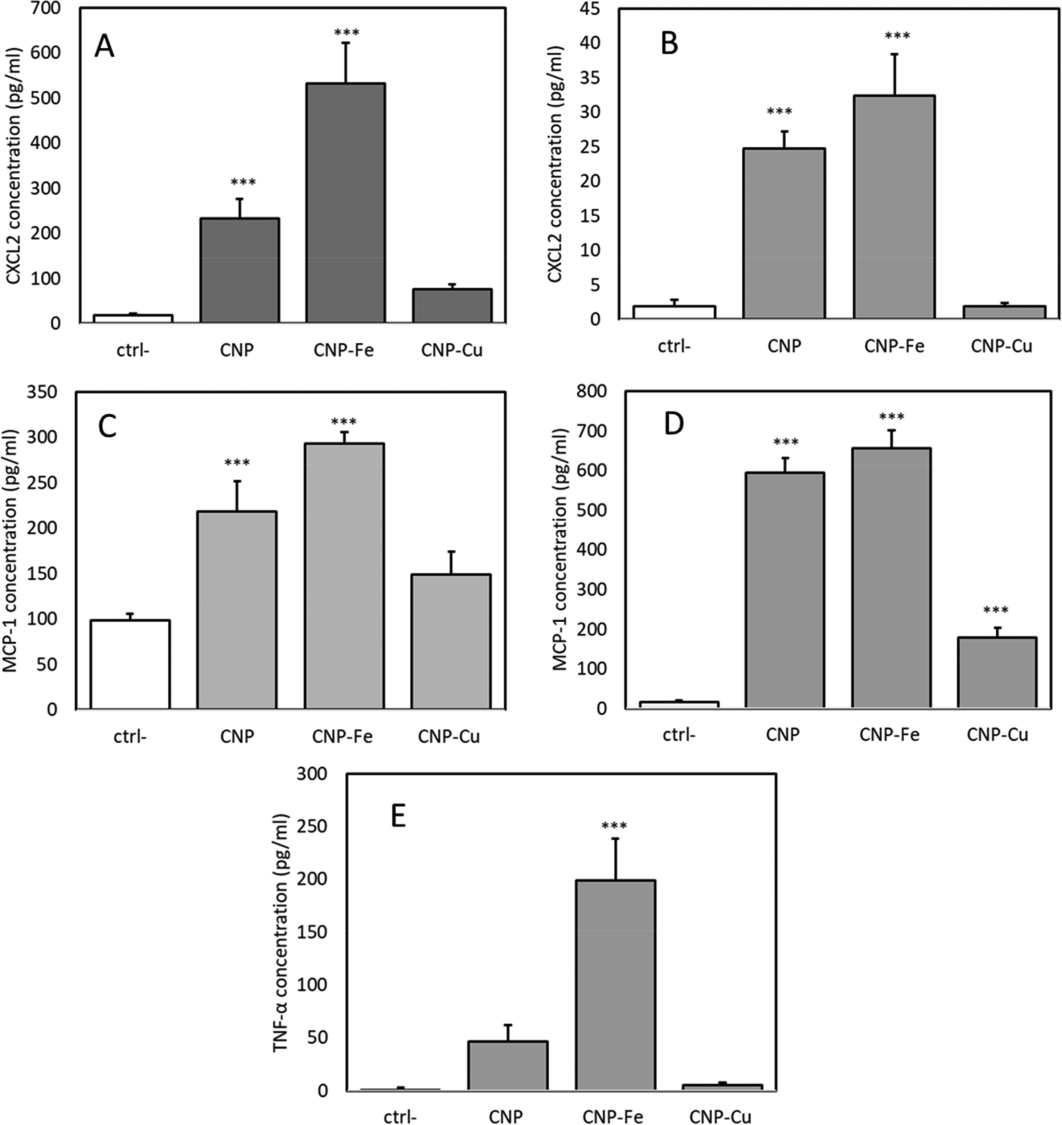
Effect of Cu and Fe on pro-inflammatory cytokine release by CNPs. (A and B) CXCL2, (C and D) MCP-1, and (E) TNF-α release from NR8383 macrophages (A, C, and E) and RLE epithelial cells (B and D) following exposure to pristine or loaded CNPs (5 μg/cm^2^). ** *p* < 0,05, *** *p* < 0.01 vs ctrl (0 μg/mL).

To monitor the ability of NPs to initiate pro-inflammatory responses, the secreted levels of the cytokine TNF-α and the chemokines MCP-1 and CXCL2 were measured in the supernatants of the RLE and NR8383 cells following 24 h exposure to CNP, CNP–Fe, and CNP–Cu at 5 μg/cm^2^ (Figure 8).

The secretion of MCP-1 and CXCL2 from both cell lines and of TNF-α in the NR8383 cells was observed for pristine and Fe-loaded CNPs. In most cases, the effects of the Fe-loaded CNPs tended to be stronger than those the pristine CNPs. The effect of Cu–CNP was much lower in both cell lines and only statistically significant for MCP-1 release from the RLE epithelial cells.

## Discussion

Environmental and traffic policies are generally based on regulated components of PM (PM10 and PM2.5) or gaseous substances like NOx. However, other components having documented health effects like carbon black or transition metals like Cu and Fe or Zn were proposed as relevant indicators of PM-associated pathogenicity.^36^ The results of the present study support this suggestion, indicating that copper and iron ions, and in particular the fraction of ions bound to the NP surface, play a key role in the OP of ultrafine PM and their ability to cause oxidative stress, inflammatory cytokine release, and DNA damage in lung cells.

### Contribution of Cu, Fe, and Carbonaceous Particles to the Surface Reactivity of PM

Fe and Cu are redox-active metals, able to generate ROS by Fenton chemistry or by reduction of oxygen, a reactivity that depends upon their coordinative and oxidation state. On the other hand, elemental carbon particles are not inert, as they have been reported to have both antioxidant^27−29^ and pro-oxidant^30,31^ properties. Both components can therefore contribute to the oxidative potential of PM. Our data indicate that both pristine and loaded CNPs had a significant scavenging activity toward the most aggressive ROS, hydroxyl radicals. However, in the case of CNP–Cu, the scavenging activity was lower, suggesting the existence of two separate mechanisms, the generation of hydroxyl radicals by copper ions and the scavenging activity of the carbon nanoparticles. In fact, CNP–Cu generate a large number of hydroxyl radicals in the presence of H_2_O_2_. On the other hand, the concentration of radicals generated was lower than that generated by free Cu(II), even though the copper concentration was the same. This difference could be again explained by assuming that the carbonaceous particles act as scavengers of hydroxyl radicals or, alternatively, by a reactivity of bound ions lower than the free ones.

The mechanism of the copper-induced Fenton-like reaction is still under discussion. The main mechanism proposed includes the reduction of Cu(II) by H_2_O_2_ (reactions 1 and 2) with the formation of O_2_^–•^ and Cu(I), the latter able to react with H_2_O_2_ to form HO^•^ (reaction 3), albeit the possible involvement of Cu(III) as a reaction intermediate has been also hypothesized.^37,38^
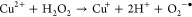
1
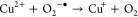
2
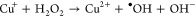
3In the present case, copper ions are reduced by the particles, a process that likely occurs also in PM, thus contributing to their OP. A similar mechanism was expected with iron. However, both CNP–Fe and soluble iron ions generate a smaller amount of hydroxyl radicals from H_2_O_2_ than copper. These results are in agreement with what was previously found by Shi and co-workers.^23^ In this study, the Fenton reactivity of carbon black samples loaded with a different metal was investigated.^23^ Similarly, the presence of copper was associated with a Fenton reactivity higher than that for iron.

The higher reactivity of Cu with respect to Fe should be ascribed to the possible generation of a more efficient oxidation–reduction cycle by copper with respect to iron, due to the reduction potential of the couple Cu(II)/Cu(I) (*E*° = +0.16 V) being closer to the SHE potential than the couple Fe(III)/Fe(II) (*E*° = +0.77 V). A different coordination state of the two ions at the surface of the particles modifying their reduction potential can also explain the reason for their different reactivity.^39^ In particular, Fe(III) has a high tendency to form oxo–hydroxides even at acidic pH: the presence of these species on CNPs might also explain its lower involvement in redox cycles with respect to Cu.

Several studies have attempted to correlate the amount of metals with the OP of PM. However, by measuring the OP by, e.g., dithiothreitol (DTT) assay or macrophage ROS assay,^40^ a poor correlation was found in real PM samples.^36^ Our data suggest that this poor correlation might be due to differences in the speciation of metals or in a variability in the carbonaceous core scavenging capacity. In fact, previous studies showed that the antioxidant activity of carbon nanoparticles depends upon the crystallinity of the carbon framework.^27,29^

The interaction of nanoparticles with biological fluids and with the media used in cellular tests has been previously shown to modify the toxicological outcome,^41−43^ an effect strictly dependent upon the kind of material.^44^ One of the main processes is the formation of a protein corona masking the surface.^45^ As a consequence, a decrease in the surface reactivity^44^ or a modification of the size distribution^46^ may occur. On the other hand, the exchange of redox-active ions between the nanoparticle and the medium may affect their oxidative potential. In the present case, both pristine and loaded CNPs exhibit in the medium a reactivity similar to that observed in water. The reactivity of CNP–Cu in the cell medium was slightly lower than that in water, in agreement with the lower amount of bonded ions due to the leaching occurring in the medium. On the other hand, leached ions lose their reactivity, likely because of the presence of chelating species able to modify their redox potential. At the same time, the size distribution of pristine and loaded CNPs remains similar to water, except for CNP–Cu that slightly agglomerated in the cell medium.

### Copper and Iron Modulate the Toxicity of PM toward Lung Macrophages and Epithelial Cells

Following 24 h of incubation, CNP–Cu nanoparticles induced a clear decrease in the viability of NR8383 macrophages starting from 10 μg/cm^2^ in a dose-dependent manner. This result is in agreement with a previous study^47^ in which the coexposure of carbon black with Cu(II) ions induced an increase of the cytotoxicity on RAW264.7 macrophages.

No pronounced toxic effect was observed for CNP and CNP–Fe up to 80 and 40 μg/cm^2^, respectively. On the other hand, these NPs unexpectedly induced and increased the WST-1 activity. This increase of viability may be explained by an activated state of the cells resulting from CNP treatment, leading to a stronger activation of the mitochondria and thus to an increased activity of WST-1 reducing dehydrogenases. Increased mitochondrial activity could be an indicator of hormesis, which is an activation of cells in order to adapt to the external stimuli.^48^ Note that an increase of the metabolic activity of human hepatocellular carcinoma cell line was previously observed after treatment with graphene oxide and carboxyl graphene nanoplatelet.^49^ In the study, the authors suggested a mechanism based on the ability of these materials to induce plasma membrane mechanical damage. Such damage is supposed to initiate energy-dependent processes involved in plasma membrane repair, enhancing the metabolic activity.^49^ Note, however, that graphene is morphologically different from CNPs, which are round shaped and are not expected to cause cell membrane damage, as previously shown by some of us, suggesting the occurrence of an alternate pathway.^29^

The increase of mitochondrial activity after particles treatment was not observed on RLE epithelial cells. In this case, treatment with the pristine nanoparticles induced a slight decrease of viability, while the presence of Fe and Cu largely increased the toxicity of the CNPs, similar to what was observed in macrophages, with a major effect for Cu. CNP–Cu also induces damage to the cell membranes with an effect more evident on macrophages than on epithelial cells. This is consistent with a possible higher uptake of the particles by macrophages with respect to epithelial cells.

Albeit a substantial fraction of Cu(II) ions was released in the medium during incubation, CNP–Cu showed higher toxicity toward macrophages than free ions at the same concentration, suggesting that internalization occurred faster than release or alternatively that the small amount of ions remaining at the surface is enough for the toxic effect observed.

The effect of copper and iron on the induction of oxidative stress by CNPs was also evaluated by monitoring the Nrf2 translocation in the nucleus. This redox-sensitive transcription factor is well known for its protective role in oxidant- and particle-induced lung disease,s^50^ and its activation in cells has been promoted as a sensitive marker of the toxicity of ultrafine PM.^51^ All of the particles induced Nrf2 translocation, but CNP–Cu-treated cells showed a stronger effect than treatment with CNP and CNP–Fe, especially in the RLE cells. This result correlates well with the high direct OP of CNP–Cu assessed by cell-free experiments, strongly supporting a mechanism of toxicity of Cu-loaded CNPs driven by particle-derived ROS.

Moderate DNA damage was detected for all nanoparticles, but CNP–Fe and CNP–Cu caused stronger DNA damage than the pristine nanoparticles after a treatment concentration of 40 μg/cm^2^. In all cases, free ions caused lower DNA damage than the corresponding loaded nanoparticles. Nuclear penetration of the particles has been discussed as a prerequisite causing direct genotoxicity.^52,53^ However, due to the size of the CNPs, a mechanism whereby nanoparticles enter the nuclei upon translocation through the pores of the nuclear membrane may be excluded. This suggests an indirect pathway that involves induction of oxidative stress. The higher DNA damage observed with the metal-loaded CNPs could be due to a Trojan Horse mechanism whereby intracellularly the metals could reach the DNA upon release from the particles in the cytoplasm. In this study, the highly sensitive alkaline comet test allowed us to identify clear differences in the degree of DNA damage depending on metal loading of the nanoparticles. To further elaborate on possible underlying mechanisms and consequences, it will be interesting to explore DNA damage-associated responses, like induction of DNA repair, cell cycle arrest, or apoptosis.^54,55^

The increase of expression of pro-inflammatory cytokines is a common end point that indicates the activation of macrophages and epithelial cells. Tumor necrosis factor-α (TNF-α) is a potent early pro-inflammatory cytokine that plays a key role in many inflammatory lung diseases, while CXCL2 is a potent chemoattractant factor for neutrophils^56^ that are accumulated in the lung after inhalation to toxic particles, playing an important role in tissue damage. MCP-1 (monocyte chemoattractant protein-1) is a potent chemotactic factor for monocytes, and it is released by a variety of cell types after induction by oxidative stress, cytokines, or growth factors.

Significant release of cytokines was observed after treatment with pristine and loaded CNPs. The trend was similar for all cytokines and cell lines, i.e., CNP–Cu < CNP < and CNP–Fe.

In Table 2 a summary of the results is reported. Both iron and copper ions appear to exacerbate the toxicity of CNPs; however, while Fe largely increases the NP-induced release of pro-inflammatory cytokines, having a minor effect on viability, copper increases the NP cytotoxicity and showed more pronounced activation of the oxidative stress-activated transcription factor Nrf2. DNA damage in epithelial cells was increased to a similar extent for the copper and iron loading. Overall, the present data confirm the role of Fe and Cu as determinant of the toxicity of PM as previously suggested by Guastadisegni et al. in 2010.^57^ In this study, a clear correlation between Fe and Cu in PM samples collected in different sites and their pro-inflammatory effect on RAW264.7 macrophages was reported. More importantly, Schaumann et al. found in 2004 a clear correlation between the amount of Cu and Zn in PM2.5 with lung inflammation in humans.^58^

**Table 2:** Summary of the Effect of Iron and Copper on CNP Toxicity

model	end point	CNP	CNP–Fe	CNP–Cu
cell free	ROS	–	+	++
macrophages	WST-1	(−)^a^	(−)^a^	+++
	LDH	–	+	++
	Nrf2	+	+	+++
	CXCL2	+	+++	–
	MCP-1	++	+++	+
	TNF-α	–	+++	–
epithelial cells	WST-1	--	+	+++
	LDH	+	+	+++
	DNA damage	+	++	++
	Nrf2	+	++	+++
	CXCL2	+++	+++	–
	MCP-1	+++	+++	+

a Proliferative effect.

## Conclusions

In conclusion, the data reported herein show that bound Cu and Fe ions are important determinants of toxicity of PM toward lung cells. Moreover, a clear correlation between the cell-free oxidative potential and the effect on macrophages and epithelial cells was observed for Cu but not for Fe, suggesting the existence of different mechanisms of action for the two ions. Finally, OP of PM appears to be influenced by the carbonaceous component of PM that acts as free radical scavenger. This may possibly contribute to the variability of the OP of real PM samples.

## Supporting Information Available

Mean hydrodynamic diameter of pristine and loaded CNPs in water and cell medium; TEM image of pristine and loaded CNP and EEL/ESI analysis of CNP–Fe; effect of CNPs on the oxidative state of iron and copper; comparison of the Fenton reactivity of aqueous copper and iron ions vs ions bonded to CNP; oxidative potential of copper ions in cell culture medium; size distribution change of pristine and loaded CNPs in cell medium; comparison between the effect of aqueous copper and iron ions vs ions bonded to CNPs on cytotoxicity in macrophages and epithelial cells (PDF)

## Abbreviations

CNP: carbon nanoparticles
EPR: electronic paramagnetic resonance
NP: nanoparticles
OP: oxidative potential
PM: particulate matter
