# Supplementary material for: Mechanistic Insights into the Role of Iron, Copper, and Carbonaceous Component on the Oxidative Potential of Ultrafine Particulate Matter
Source: Chem Res Toxicol. 2021 Mar 2;34(3):767–79. doi: 10.1021/acs.chemrestox.0c00399 (PMC8034814; doi:10.1021/acs.chemrestox.0c00399)
Supplement: Supplementary file 1 — tx0c00399_si_001.pdf [file tx0c00399_si_001.pdf]

Mechanistic insights into the role of iron, copper and carbonaceous component on the oxidative potential of ultrafine particulate matter.

Ion Tacu [‡,§], Ida Kokalari[‡], Ornella Abollino[‖], Catrin Albrecht[§,†], Mery Malandrino[‡], Anna Maria Ferretti[°], Roel P.F. Schins[§], Ivana Fenoglio[‡*]

[‡]*Dept. of Chemistry, University of Torino, 10125-Torino, Italy*

[§]*IUF - Leibniz Research Institute for Environmental Medicine, 40225-Düsseldorf, Germany*

[‖]*Dept. of Drug Science and Technology, University of Torino, 10125-Torino, Italy*

° *Istituto di Scienze e Tecnologie Chimiche "Giulio Natta" SCITEC CNR, Via Fantoli 16/15 20138 Milan, Italy;*

† *Current address: State Office for Consumer Protection Saxony-Anhalt, Stendal, Germany*

Supporting Information

Table S1

Mean hydrodynamic diameter (Z-average) and PDI (% intensity)

H_2O			cell culture medium			
	0 h		0 h		24 h	
	dH (nm)	PDI	dH (nm)	PDI	dH (nm)	PDI
**CNP**	182,1±4,3	0,098 ± 0,05	178,3 ± 2,5	0,0765 ± 0,05	180,5 ± 0,8	0,098 ± 0,07
**Fe-CNP**	172,1 ± 0,8	0,092 ± 0,06	168,9 ± 1,2	0,072 ± 0,003	170,9 ± 0,8	0,073 ± 0,01
**Cu-CNP**	195,1 ± 3,4	0,092 ± 0,001	300,2 ± 6,2	0,102 ± 0,004	326,5 ± 11,1	0,1515 ± 0,03

Mean hydrodynamic diameter (% number)

	H_2O	Cell culture medium	
	dH (nm)	dH (nm) 0h	dH (nm) 24h
**CNP**	166.9 ± 6.3	164.2 ± 5.9	164.4 ± 11.5
**Fe-CNP**	153.8 ± 7.6	154.7 ± 6.8	155.3 ± 3.5
**Cu-CNP**	180.5 ± 5.9	289.9 ± 5.4	307.2 ± 9.0

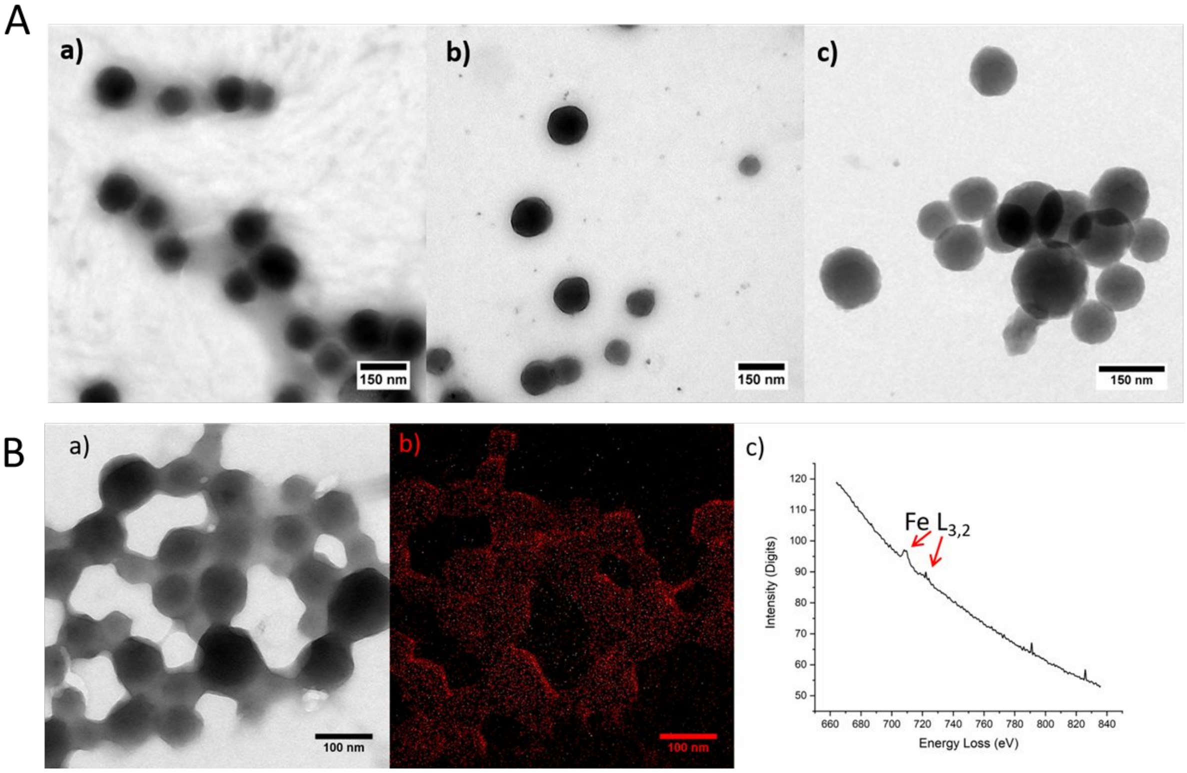

Figure S1. (A) Representative TEM images of a) pristine CNP; b) CNP-Fe; c) CNP-Cu; (B) a) TEM image of the CNP-Fe sample, b) ESI map of the iron in the CNP-Fe sample, c) EELS spectrum of the CNP-Fe sample.

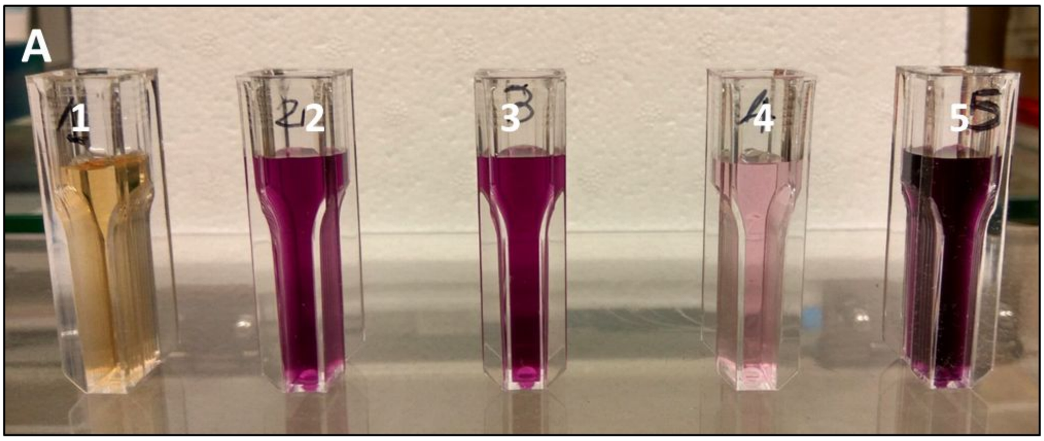

	1	2	3	4	5
$Fe(NO_3)_3$		X		X	X
CNP	X	X	X	X	
Ferrozine	X	X	X		X
Ascorbic acid			X		X

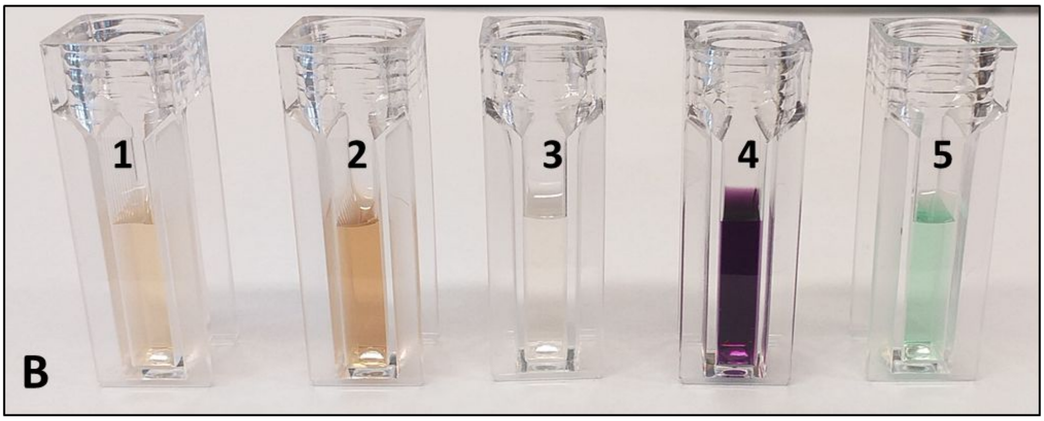

	1	2	3	4	5
$CuSO_4$			X	X	X
CNP	X	X	X	X	
Bicinchoninic acid		X		X	X

Figure S2. Effect of CNPs on the oxidative state of A) iron and B) copper; The supernatant of CNPs suspended in various solutions are shown.

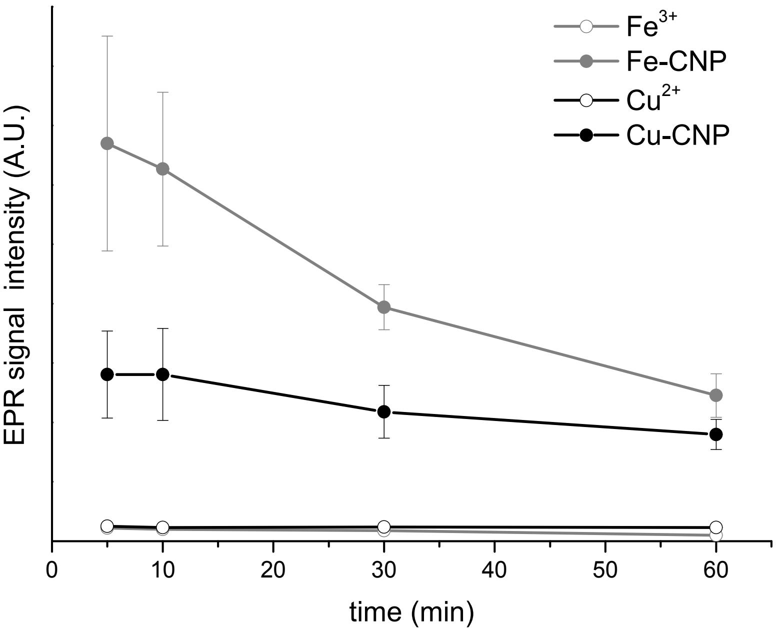

Figure S3. Comparison of the Fenton reactivity of aqueous copper and iron ions vs ions bound to CNPs. Intensity of the EPR signal obtained following incubation of loaded CNPs (0.30 mg/mL) or $CuSO_4$/$FeSO_4$ in equivalent concentration in a solution containing DMPO 60.3 mM, H_2O_2 27 mM in PBS 13.3 mM, pH 7.4

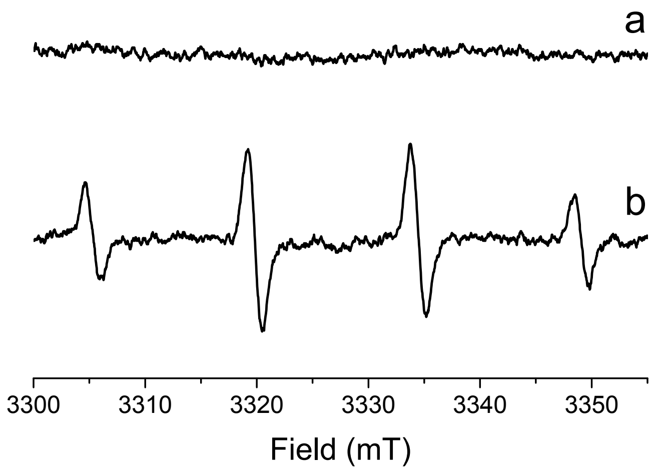

Figure S4. Oxidative potential of copper ions in cell culture medium water. EPR signal obtained by a solution in a) F-12 Ham containing 1 % of glutamine, 1% with penicillin-streptomycin and 5% FBS **(**0.128 µg/ml **)**; and b) ultrapure water of $CuSO_4$ ($9.26x10^{-5}$ M) in the presence of DMPO (60.3 mM) and H_2O_2 (27 mM) in PBS 13.3 mM, pH 7.4

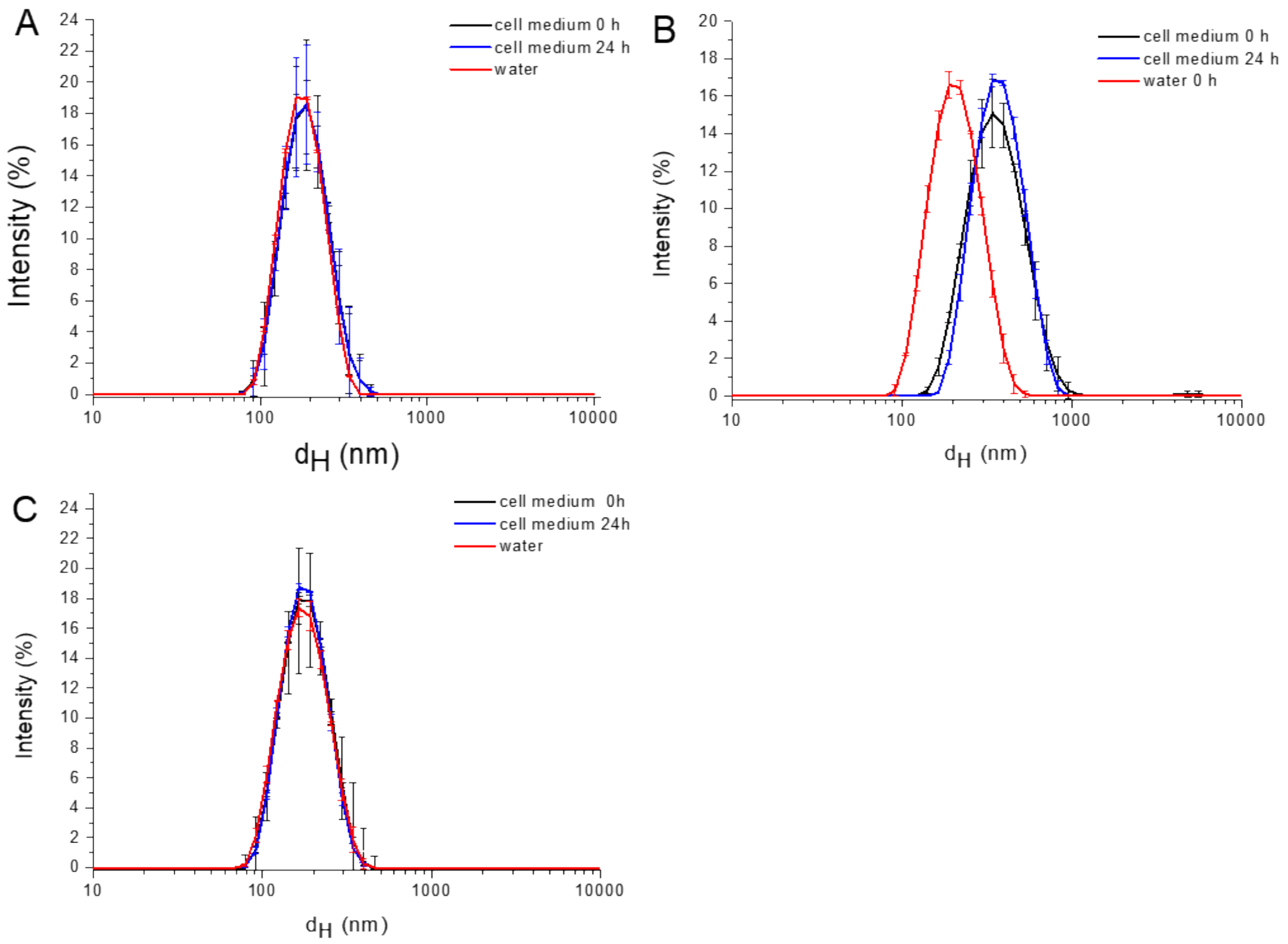

FIGURE S5. Size distribution of pristine and loaded CNPs in cell medium. Hydrodynamic diameters distribution (% intensity) of A) CNP; B) CNP-Cu; C) CNP-Fe in F-12 Ham containing 1 % of glutamine, 1% with penicillin-streptomycin and 5% FBS (0.128 μg/ml) measured by DLS after 0 h and 24 h of incubation at 37°C compared with that in water.

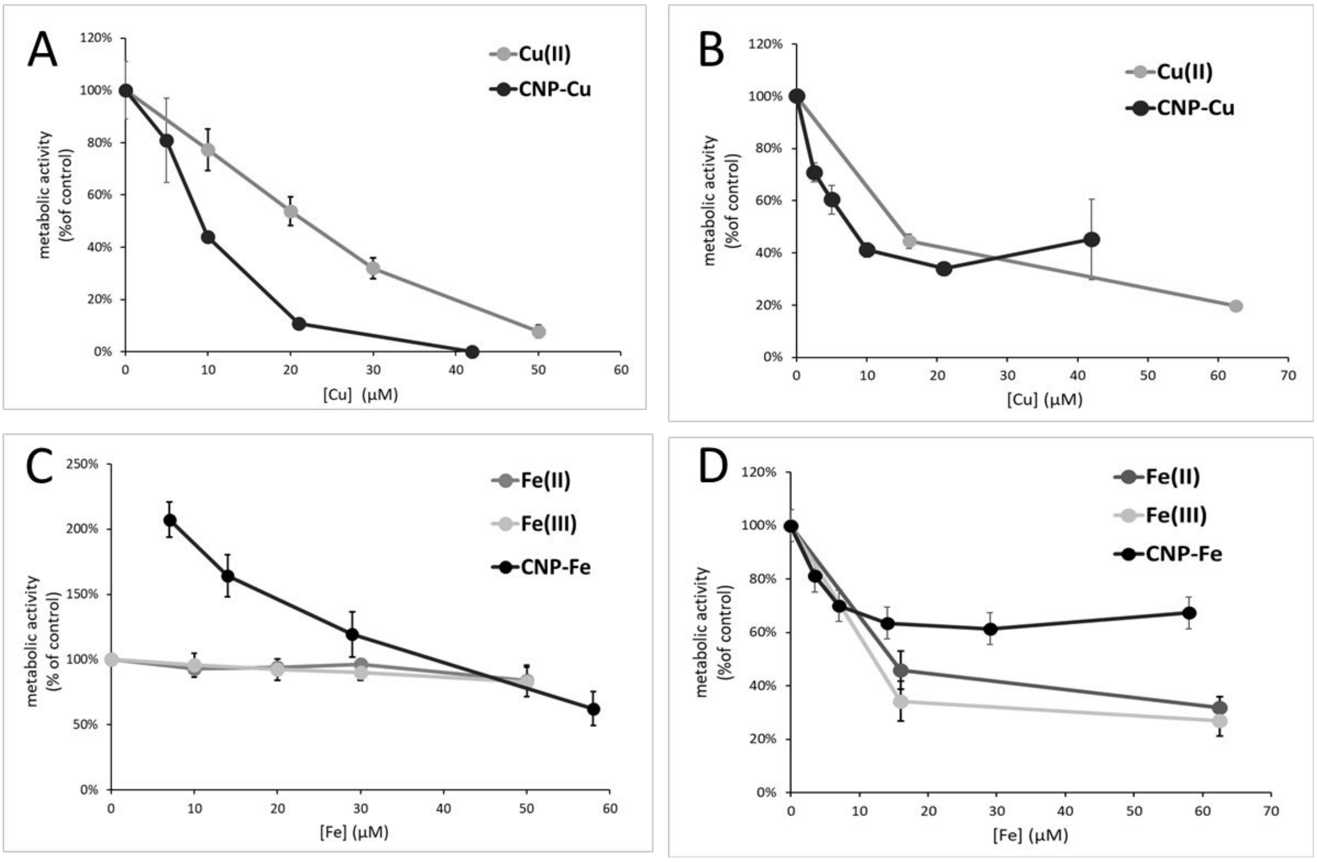

Figure S6. Comparison between the effect of aqueous copper and iron ions vs ions bounded to CNP on the metabolic activity (WST-1) of A,C) macrophages; and B,D) epithelial cells.
